# Increased Plasma YKL-40/Chitinase-3-Like-Protein-1 Is Associated with Endothelial Dysfunction in Obstructive Sleep Apnea

**DOI:** 10.1371/journal.pone.0098629

**Published:** 2014-05-30

**Authors:** Behrouz Jafari, Jack A. Elias, Vahid Mohsenin

**Affiliations:** 1 Section of Pulmonary, Critical Care and Sleep Medicine, University of California, Irvine, California, United States of America; 2 Warren Alpert School of Medicine, Providence, Rhode Island, United States of America; 3 Section of Pulmonary, Critical Care and Sleep Medicine, Yale School of Medicine and 4 John B. Pierce Laboratory, New Haven, Connecticut, United States of America; The University of Manchester, United Kingdom

## Abstract

**Purpose:**

Obstructive sleep apnea (OSA) is a common disorder affecting 15–24% of the adults and is associated with increased risk of hypertension and atherosclerosis. The exact mechanisms underlying hypertension in OSA are not entirely clear. YKL-40/Chitinase-3-like protein-1 is a circulating moiety with roles in injury, repair and angiogenesis that is dysregulated in atherosclerosis and a number of other diseases. We sought to determine the role of YKL-40 in endothelial dysfunction and hypertension in OSA.

**Methods:**

We studies 23 normotensive OSA (N-OSA) and 14 hypertensive OSA (H-OSA) without diabetes and apparent cardiovascular disease. Endothelial-dependent nitric oxide-mediated vasodilatory capacity was assessed by flow-mediated vasodilation (FMD). YKL-40, vascular endothelial growth factor (VEGF) and the soluble form of VEGF receptor-1or sFlt-1 were measured in plasma using ELISA methodology.

**Results:**

N-OSA subjects aged 49.1±2.3 years and H-OSA aged 51.3±1.9 years with BMI 36.1±1.6 and 37.6±1.9 kg/m^2^, respectively. The apnea-hypopnea index (AHI) was 41±5 events/hr in N-OSA and 46±6 in H-OSA with comparable degree of oxygen desaturations during sleep. FMD was markedly impaired in H-OSA (8.3%±0.8) compared to N-OSA (13.2%±0.6, *P*<0.0001). Plasma YKL-40 was significantly elevated in H-OSA (55.2±7.9 ng/ml vs. 35.6±4.2 ng/ml in N-OSA, *P = *0.02) and had an inverse relationship with FMD (r = −0.52, *P* = 0.013). There was a significant positive correlation between sFlt-1/VEGF, a measure of decreased VEGF availability, and YKL-40 (r = 0.42, *P = *0.04).

**Conclusion:**

The levels of plasma YKL-40 were elevated in H-OSA group and inversely correlated with the endothelial-dependent vasodilatory capacity whereas there was a positive correlation between sFlt-1/VEGF and YKL-40. These findings suggest that YKL-40 is dysregulated, in part, due to perturbation of VEGF signaling, and may contribute to endothelial dysfunction and hypertension in OSA.

## Introduction

Obstructive sleep apnea (OSA) is a highly prevalent sleep disorder that affects 15–24% of the adults and is associated with increased cardiovascular morbidity and mortality including hypertension [1]. OSA is characterized by recurrent upper airway obstruction during sleep. These episodes are terminated by arousals and are commonly associated with hypoxemia. Current evidence suggests that inflammatory processes, oxidative stress and endothelial dysfunction may play roles in the pathogenesis of vascular complications of OSA [2]. Endothelial dysfunction is the earliest event in atherosclerosis, and plays a pivotal role in all phases of the atherosclerotic process from the initiation of the fatty streak to plaque rupture [3], [4]. Vascular endothelial growth factor (VEGF) plays a significant role in angiogenesis and endothelial function [5], [6]. It is primarily regulated via hypoxia-inducible factor-1α (HIF-1α) in response to hypoxia and its circulating level is increased in patients with OSA [7]. We have previously shown that hypertensive OSA patients have increased circulating levels of VEGF receptor-1 also known as sFlt-1 compared to normotensive counterparts [8]. Circulating sFlt-1 is a spliced soluble variant of Flt-1, which binds to VEGF. While sFlt-1 is not a vasoconstrictor, it does significantly inhibit the vasodilatory actions of VEGF *in vitro*, and chronic elevations in circulating concentrations result in increased blood pressure [9]–[11].

YKL-40 is a 40 kDa heparin- and chitin-binding glycoprotein also known as human cartilage glycoprotein 39 or chitinase-3-like protein-1 (CHI3L1) [12]. YKL-40, a member of ‘mammalian chitinase-like proteins’ is a phylogenetically highly conserved serum protein with homologues in vertebrates and invertebrates. Though YKL-40 contains highly conserved chitin-binding domains; it functionally lacks chitinase activity and its cell surface receptor was recently described as requiring an IL-13R α2-dependent mechanism for its effector responses [13]. YKL-40 is, in part, regulated by VEGF and is secreted by several cell types of the innate immune system, epithelial cells and by differentiated vascular endothelial cells and vascular smooth muscle cells [14]. YKL-40/Chitinase-3-like protein-1 is a circulating moiety with roles in antipathogen responses, injury, repair and angiogenesis that is dysregulated in atherosclerosis and a number of other diseases. YKL-40 protein expression is found *in vivo* in both macrophages and human smooth muscle cells in atherosclerotic plaques [15].

YKL-40 dysregulation often correlates with the severity and natural history of cardiovascular disorders. Elevated plasma YKL-40 level is associated with increased risk of ischemic stroke [16], [17] and found to be a biomarker for myocardial infarction, progression of coronary artery disease, congestive heart failure [18], and cardiovascular death [19], [20]. OSA has been shown to increase population burden of cardiovascular diseases including stroke and hypertension [21], [22]. The relative risks for the development of incident coronary artery disease, stroke or hypertension are in the order of 3-fold over several years [21]–[25]. However, these epidemiologic studies do not identify individuals at risk. The biomarkers that predict the development or severity of vascular pathology in OSA have not been validated nor are the pathogenetic mechanisms that engender this vascular response understood. Two-third of patients with moderate to severe OSA has hypertension while the others remain normotensive despite exposure to severe intermittent hypoxia during sleep [26]. The mechanism(s) underlying this divergent phenotype is poorly understood. The role of YKL-40 in endothelial function and hypertension in OSA is not known. In view of aforementioned reports on the role of YKL-40 in cardiovascular diseases we hypothesized that YKL-40 might be abnormal and play a role in endothelial dysfunction and hypertension in patients with OSA. To test this hypothesis we quantified plasma levels of YKL-40 in OSA patients matched for age and co-morbidities with and without hypertension and compared these values to measures of endothelial function by assessing flow-mediated nitric oxide-dependent vasodilatory capacity. Our study demonstrates that YKL-40 is elevated in hypertensive OSA patients and correlates inversely with measures of endothelial function. Further, our study provides mechanistic insights by highlighting a positive relationship between sFlt-1/VEGF, a measure of decreased free VEGF, and YKL-40 in OSA.

## Methods

### Subjects

Patients were recruited consecutively from among those screened for sleep-disordered breathing at Yale Center for Sleep Medicine. Patients with newly diagnosed and untreated moderate to severe OSA (apnea-hypopnea index, AHI ≥20 events/hr) with and without hypertension were enrolled. The subjects are a subset of a cohort that has been published previously [27]. Hypertension was defined by blood pressure ≥140 mm Hg systolic and/or ≥90 mm Hg diastolic, which had been previously documented by using appropriate sized cuff and measurements that had been made at least in three different occasions according to the standard criteria [28]. Subjects were excluded if they had diabetes mellitus, chronic kidney disease, peripheral vascular disease, liver disease, hemolytic anemia, inflammatory disease, active infection, or if they were pregnant, on therapy for OSA, on chronic steroid treatment, or younger than 18 years of age. Each subject was informed of the experimental procedures and signed the consent form for this study that had been approved by the Human Investigation Committee of the Yale University School of Medicine.

### Sleep Study

Nocturnal polysomnography was performed as previously described [8]. Respiratory events were scored according to the American Academy of Sleep Medicine. Hypopnea was scored when there was at least 30% decrease in airflow signal with a ≥4% decrease in oxygen saturation. Oxygen desaturation index (ODI) was defined as the number of oxygen desaturation of ≥4% per hour sleep. The percentage of total sleep time associated with oxyhemoglobin saturation of <90% (T<90%) was calculated as a measure of hypoxemia duration.

### Endothelial Function

Endothelial function was assessed by a standard flow-mediated vasodilation (FMD) method using Doppler ultrasound of the brachial artery between 9 am and 12 noon as previously described [27]. Subjects had refrained from smoking or consumption of caffeine-containing beverages and fasting for 12 hours prior to the study. FMD was expressed as the percentage of change in the brachial artery diameter from baseline to following peak reactive hyperemia after cuff deflation. The artery diameters were measured independently by two investigators (one blinded to grouping of subjects) using a digital caliper and were verified by an automated border recognition software. Peak vasodilation was calculated as the percent change in the brachial artery diameter from baseline to peak reactive hyperemia. The inter-observer and intra-observer variability in diameter measurements were less than 5%.

### Blood Sample

Venous blood sample was obtained after the subjects had been seated and rested for 60 min between 9 am and 12 noon after a 12-hour fast. Plasma was separated with centrifugation at 1200 g for 10 min at 4°C, aliquoted and stored at −80°C for further analysis.

### YKL-40 Assay

Measurement of plasma YKL-40 levels was performed in duplicate with the use of commercially available enzyme-linked immuno-sorbent assay (ELISA) kits (Quidel Corporation, San Diago, CA). The minimum detection limit of the YKL-40 assay is 5.4 ng/ml. Inter- and intra-assay coefficient of variations for the assays were <7%.

### Measurement of VEGF and sFlt-1

Plasma VEGF levels were determined in duplicates using ELISA kit from Abnova (Walnut, CA) with the sensitivity of 5 pg/ml. Circulating levels of sFlt-1 in plasma were measured using commercially available reagents and recombinant standards (R&D Systems, Minneapolis, MN, USA). All samples were assayed in duplicate. Standards and control samples were run simultaneously for validation. The minimum detection limit for sFlt-1 was 3.5 pg/ml. Inter- and intra-assay coefficient of variations for the assays were <10%. The assay kit measures total plasma sFlt-1.

### Data Analysis

The primary outcome was endothelial-dependent vasodilation as measured by FMD. The required sample size to detect a significant change in FMD (delta  = 4, SD = 2.7) was 14 per group (alpha = 0.05, power = 80%). However, we over sampled the normotensive OSA group to account for the differential individual responses to OSA and hypoxia. Data were analyzed using Student *t*-test for comparisons of the groups. Spearman correlation was used to analyze the relationship between FMD and YKL-40 and other variables. Data were analyzed using Graphpad Prism (La Jolla, CA). Z-test was used for comparison of percentages for categorical variables. Data are expressed as means ±SE. *P* values were 2-sided with a level of significance of *P*<0.05.

## Results

### Subjects Characteristics

The subjects had severe OSA and groups were comparable in terms of AHI and exposure to hypoxia (ODI and T<90%) during sleep (Table 1). They were matched for age, body mass index (BMI) and co-morbidities with no significant difference in gender distribution between groups.

**Table 1:** Subjects’ characteristics.

	OSA	
	Normotensive	Hypertensive
	(n = 23)	(n = 14)
Age, yr	49.1±2.3	50.6±2.0
Male, %	74	86
BMI, kg/m^2^	36.1±1.6	37.6±1.9
Smoker, %	17	35
Dyslipidemia, %	30	29
AHI, events/hr	41±5	46±6
ODI >4%/hr	33±6	38±7
T<90, min	35±9	52±12
Nadir SaO_2_, %	76±2	79±1

BMI, body mass index; AHI, apnea-hypopnea index; ODI, oxygen desaturation index; T<90, total sleep time in minutes with oxygen saturation <90%; Data are means ± SE. There are no significant differences in the above parameters between the groups.

### Endothelial-dependent Flow-Mediated Vasodilation (FMD) and YKL-40

**Figure 1:**
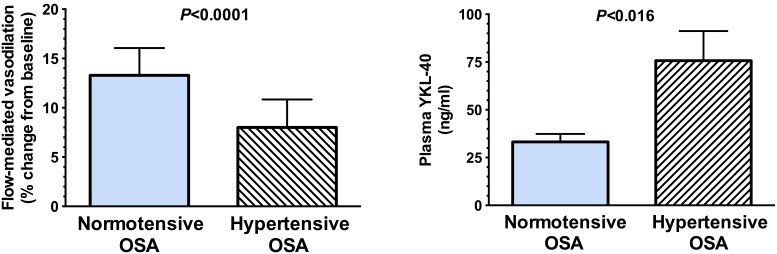
Endothelial-dependent nitric oxide-mediated vasodilatory capacity and Plasma YKL-40. Hypertensive OSA patients had marked impairment in flow-mediated vasodilation compared with normotensive OSA. Plasma levels of YKL-40 in hypertensive OSA were significantly higher than the normotensive OSA subjects.

**Figure 2:**
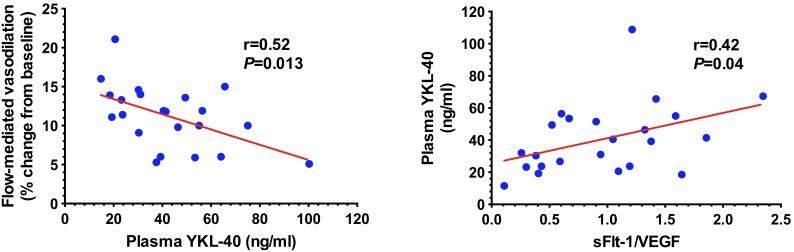
Plasma YKL-40 and sFlt-1/VEGF. YKL-40 correlated inversely with flow-mediated vasodilation whereas there was a positive correlation between YKL-40 and sFlt-1/VEGF, a measure of decreased free VEGF availability or anti-angiogenic state.

FMD was markedly impaired in hypertensive OSA (8.3%±0.8) compared to normotensive OSA (13.2%±0.6, *P*<0.0001), Figure 1. In view of the groups being tightly matched for apnea severity and hypoxia exposure we did not anticipate any correlation between FMD and the respiratory indices. Plasma YKL-40 was significantly elevated in hypertensive OSA compared to normotensive OSA (Figure 1). There was a significant inverse correlation between YKL-40 and FMD (r = −0.52, *P* = 0.013) (Figure 2).

### VEGF and sFlt-1

Plasma levels of VEGF were 54.9±8.5 pg/ml in normotensive and 47.7±4.7 pg/ml in hypertensive OSA. Plasma concentrations of sFlt-1 were 66.6±9.1 pg/ml in normotensive and 58.5±48.3 pg/ml in hypertensive OSA. sFlt-1/VEGF, a measure of decreased free VEGF, had a significant positive correlation with YKL-40 (r = 0.42, *P* = 0.04) (Figure 2). This meant that inhibition of VEGF by its circulating spliced receptor variant, sFlt-1, was associated with higher YKL-40. In an analysis of co-variance with FMD as a dependent variable and YKL-40 as an independent variable with sFlt-1/VEGF as a covariate showed significant difference in FMD between the hypertensive and normotensive group (*P* = 0.038).

## Discussion

In this study, we have demonstrated impairment in endothelial-dependent nitric oxide-mediated vasodilatory capacity in hypertensive OSA patients with significant elevation of plasma levels of YKL-40 compared with normotensive OSA. The two OSA groups were comparable for age, BMI, co-morbidities, sleep apnea severity (AHI) and hypoxia exposure indices. This divergent phenotypic response among OSA population suggests varying individual susceptibility to sleep apnea and intermittent hypoxia.

Growing clinical evidence has indicated that aberrant expression of YKL-40 is largely associated with endothelial dysfunction [14], [29]–[31] and poor cardiovascular outcome [19], [20], suggesting that serum levels of YKL-40 can serve as a diagnostic and prognostic biomarker. Middle aged OSA patients without clinical evidence for cardiovascular disease have premature atherosclerosis [23] and endothelial dysfunction predicts development of *de novo* hypertension in post-menopausal women [32].

YKL-40 is involved in activation of vascular endothelial cell and stimulation and migration of vascular smooth muscle cells [33]. YKL-40 is regulated, in part, by VEGF which binds to two tyrosine membrane receptors, VEGFR-1 (known as Flt-1) and VEGFR-2. VEGF promotes vasodilation by inducing nitric oxide and prostacyclin synthesis in endothelial cells. Transfection of U87 glioma cells with short-interfering RNAs (siRNAs) targeting all isoforms of VEGF resulted in highest up-regulation of CHI3L1 (YKL-40) and the related CHI3L2 genes [34]. Moreover, neutralization of VEGF activity in U87 cells with an anti-VEGF antibody induced up-regulation of YKL-40 [35]. Our data are consistent with these reports in view of the fact that plasma levels of YKL-40 correlated positively with sFlt-1/VEGF, suggesting that inhibition of VEGF by circulating sFlt-1 may play a role in the upregulation of YKL-40. Although molecular mechanisms underlying the induction of YKL-40 are still elusive, the levels of VEGF may be rate-limiting for YKL-40 regulation, possibly constituting a negative feedback loop. *In vivo* protein expression of YKL-40 is increased in human smooth muscle cells in atherosclerotic plaques [36] and serum levels of YKL-40 correlate with the arterial wall stiffness–another measure of endothelial dysfunction [14]. It is, therefore, conceivable to implicate YKL-40 in endothelial dysfunction and hypertension in a subgroup of patients with OSA. A more detailed phenotypic characterization of patients with OSA with assessment of endothelial function and analysis of biomarkers of inflammation including YKL-40 and angiogenic inhibitors within a larger population will be essential for stratifying the individual risks for development of hypertension and adverse cardiovascular outcome.

The study has some limitations. The majority of subjects were male, thus the results of this study cannot be generalized across genders. However, previous studies have found no difference in plasma YKL-40 levels between males and females [37] **.** Likewise, plasma YKL-40 in obese subjects without diabetes is not different from healthy lean individuals [38]. The sample size was based on the primary outcome of endothelial-dependent vasodilation and therefore the study was not powered to analyze the independent roles of angiogenic inhibitors. The cross-sectional nature of the study precludes demonstrating a causal link between YKL-40 and the hypertensive phenotype. However, the well-matched groups and exclusion of possible confounders supports the role of YKL-40 in endothelial dysfunction and hypertension in OSA.

In conclusion, elevated circulating levels of YKL-40 are associated with endothelial dysfunction and hypertension in OSA patients. The mechanism(s) involved in up-regulation of YKL-40 may, in part, be related to inhibition of VEGF by sFlt-1. YKL-40 could be a potential biomarker for endothelial dysfunction and hypertension in these patients.
