# Correction: Increased Plasma YKL-40/Chitinase-3-Like-Protein-1 Is Associated with Endothelial Dysfunction in Obstructive Sleep Apnea

**DOI:** 10.1371/journal.pone.0106395

**Published:** 2014-08-18

**Authors:** The PLOS ONE Staff

The image for Figure 1 is incorrect. Please see the correct Figure 1 here.

**Figure 1:**
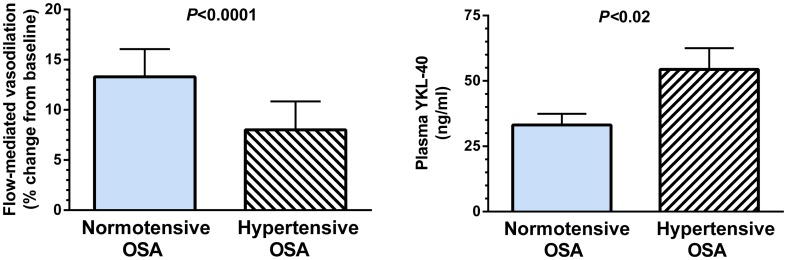
Endothelial-dependent nitric oxide-mediated vasodilatory capacity and Plasma YKL-40. Hypertensive OSA patients had marked impairment in flow-mediated vasodilation compared with normotensive OSA. Plasma levels of YKL-40 in hypertensive OSA were significantly higher than the normotensive OSA subjects.
